# Composition and Metabolic Functions of the Microbiome in Fermented Grain during Light-Flavor *Baijiu* Fermentation

**DOI:** 10.3390/microorganisms8091281

**Published:** 2020-08-22

**Authors:** Xiaoning Huang, Yi Fan, Ting Lu, Jiamu Kang, Xiaona Pang, Beizhong Han, Jingyu Chen

**Affiliations:** 1 MOE Key Laboratory of Precision Nutrition and Food Quality, College of Food Science and Nutritional Engineering, China Agricultural University, Beijing 100083, China; hxning926@sina.com (X.H.); fanyi628@126.com (Y.F.); jiamu_kang@163.com (J.K.); hbz@cau.edu.cn (B.H.); 2 Department of Bioengineering, University of Illinois at Urbana-Champaign, Urbana, IL 61801, USA; luting@illinois.edu; 3 Beijing Laboratory of Food Quality and Safety, Food Science and Engineering College, Beijing University of Agriculture, Beijing 100026, China; pxnxjc@126.com

**Keywords:** *Baijiu*, fermented grain, microbiome composition, metabolites, shotgun metagenomics

## Abstract

The metabolism and accumulation of flavor compounds in Chinese *Baijiu* are driven by microbiota succession and their inter-related metabolic processes. Changes in the microbiome composition during *Baijiu* production have been examined previously; however, the respective metabolic functions remain unclear. Using shotgun metagenomic sequencing and metabolomics, we examined the microbial and metabolic characteristics during light-flavor *Baijiu* fermentation to assess the correlations between microorganisms and their potential functions. During fermentation, the bacterial abundance increased from 58.2% to 97.65%, and fermentation resulted in the accumulation of various metabolites, among which alcohols and esters were the most abundant. Correlation analyses revealed that the levels of major metabolites were positively correlated with bacterial abundance but negatively with that of fungi. Gene annotation showed that the *Lactobacillus* species contained key enzyme genes for carbohydrate metabolism and contributed to the entire fermentation process. *Lichtheimia ramosa, Saccharomycopsis fibuligera, Bacillus licheniformis, Saccharomyces cerevisiae,* and *Pichia kudriavzevii* play major roles in starch degradation and ethanol production. A link was established between the composition and metabolic functions of the microbiota involved in *Baijiu* fermentation, which helps elucidate microbial and metabolic patterns of fermentation and provides insights into the potential optimization of *Baijiu* production.

## 1. Introduction

*Baijiu*, a traditional fermented alcoholic beverage, is very popular in China. As is the case with all naturally fermented food products, changes in the microbiome composition during fermentation are essential for the development and final quality of *Baijiu* [1,2]. Therefore, it is important to elucidate the structure and metabolic function of the microbiome so as to better understand the mechanisms underlying fermentation. Moreover, a comprehensive understanding of food microbiota may help elucidate microbial ecology and evolution in more natural, complex ecosystems [3,4,5]. Thus, there is increasing interest in uncovering the characteristics of *Baijiu*-making microbiota and revealing the important effects of changes in microbiota on *Baijiu* fermentation [1,6].

Chinese *Baijiu* can be classified into different categories based on their flavor profiles, in which sauce-flavor *Baijiu*, strong-flavor *Baijiu*, and light-flavor *Baijiu* comprise the three dominating categories [6]. Light-flavor *Baijiu* is famous for its pure, pleasantly fruity, and mild taste, and for its refreshing aftertaste [7], which have made it the predominant type of *Baijiu* in the Chinese liquor market [1,8]. Light-flavor *Baijiu* is produced in an open solid-state fermentation process. Typically, sorghum is used as raw material to which low-temperature *Daqu,* the fermentation starter for Chinese *Baijiu*, is added as culture starter, and fermentation is allowed to occur in large earthenware containers. Fermented grain is subsequently distilled to produce *Baijiu* [6]. During light-flavor *Baijiu* fermentation, microorganisms originating from *Daqu* and from the environment, such as *Bacillus*, *Lactobacillus*, *Pediococcus*, *Saccharomycopsis*, *Saccharomyces*, *Pichia*, *Wickerhamomyces,* and *Aspergillus*, were found to be predominant [9,10].

In previous studies, culture-dependent and -independent methods were used to describe the microbial community in *Daqu* and during the fermentation process of light-flavor *Baijiu* [9,10,11,12]. The functions of particular species characterized in a simulated environment differed substantially from those of species in situ [13,14]. In the past few decades, high-throughput amplicon sequencing has helped to uncover and analyze additional microorganisms that participate in *Baijiu* fermentation [6,8,9,15]. However, amplicon sequencing only provides limited information on microbial metabolic functions [16], whereas shotgun metagenomic sequencing approaches offer a higher resolution regarding taxonomic annotation, circumvent amplification biases, and provide a higher taxonomic accuracy at the species level [17,18]. Moreover, this method facilitates elucidating the functional distribution of microbiota through non-selective genomic assembly followed by gene annotation [19,20]. This approach has thus been applied in a few studies to reveal potential metabolic functions in fermented food products, including sausage [21], milk Kefirs [18], pu-erh tea [22], and various cheeses [23,24].

In the current study, shotgun metagenomic sequencing, metabolomic analyses, and gene annotation were used to investigate the microbial and metabolic characteristics during light-flavor *Baijiu* fermentation and further to assess the mechanistic correlations between microbial community composition and fermentation functions. The key microorganisms of *Baijiu* fermentation and potential metabolic functions of microorganisms were identified. Our results may help increase the production efficiency and improve the quality and flavor of the final product.

## 2. Materials and Methods

### 2.1. Sample Collection

During light-flavor *Baijiu* production, fermented grain samples were collected from Shanxi Xinghuacun *Fenjiu* Distillery Co., Ltd. (Fenyang, China) in 2017. Light-flavor *Baijiu* is fermented with sorghum as a raw material and *Daqu* as a starter culture in earthen jars. *Daqu* is a saccharifying and fermenting agent which is prepared by solid-substrate fermentation from barley and peas [11]. The diameter and depth of a jar are 0.8 and 1.2 m, respectively, with the capacity of 260 L. It takes approximately 28 days for light-flavor *Baijiu* to ferment [25]. In one fermentation batch, approximately 500 g of fermented grain samples were randomly taken from the center of the middle layer of each jar on days 1, 7, 15, and 28. The opened jars were subsequently eliminated from the study. Three independent batches were sampled for adequate representation.

### 2.2. Microbiological and Physicochemical Analysis

Changes in the physicochemical properties of fermented grain, including moisture content, pH, core temperature, and acidity, were measured as described by Pang et al. [6]. Fermented grain samples (10 g) were placed in an appropriate peptone physiological salt solution to determine the population of microorganisms using selective media. The viable cell counts of total aerobic bacteria were enumerated on Plate Count Agar at 30 °C for 24 h. Lactic acid bacteria (LAB) counts were performed on de Man, Rogosa, Sharpe Agar (MRSA) with 500 μg/mL of natamycin at 30 °C for 48 h. Yeasts and molds were enumerated on Rose Bengal Chloramphenicol Agar with 100 mg/L of chloramphenicol at 28 °C for 48–72 h [11].

### 2.3. Polar Non-Volatile Metabolite Analysis

The polar non-volatile compounds in fermented grain were assayed using the proton nuclear magnetic resonance (^1^H NMR). Metabolites were extracted by suspending 300 mg of fermented grain in 1.5 mL of cold ultra-pure water (Milli-Q; Millipore, Bedford, MA, USA), followed by grinding using a Mini-Beadbeater for 60 s and cooling on ice for 10 min. After centrifugation at 13,000× *g* for 10 min, 1.0 mL of supernatant was mixed with 1.0 mL of phosphate buffer (0.1 M sodium phosphate consisting of 10% deuterium oxide (D_2_O) (*v*/*v*), 100 mM of imidazole, 0.2% (*w*/*v*) sodium azide, and 1 mM of trimethylsilylpropionate ([Sigma-Aldrich, St. Louis, MO, USA] as an internal standard)), which was then centrifuged at 16,060× *g* and 4 °C for 5 min. Then, 600 μL of the supernatant was transferred to 5 mm NMR tubes. The NMR spectra were recorded using an Avance III 600 FT-NMR spectrometer (Bruker, Billerica, MA, USA) at 14.1 T (600.13 MHz proton frequency). The experimental NMR spectra were compared with those of known metabolites using the Chenomx NMR Suite software (version 6.0; Chenomx, Edmonton, AB, Canada) to identify the metabolites.

### 2.4. Volatile Compound Analysis

The volatile compounds in fermented grain were analyzed using an Agilent 6890 GC equipped with a 5975B series mass spectrometric detector (Agilent Technologies, Palo Alto, CA, USA). A 50:30 mm DVB-CAR-PDMS SPME fiber (Supelco Co., Bellefonte, PA, USA) was used for headspace solid-phase microextraction (SPME). Briefly, 2 g of the fermented grain sample was added to 8 mL of Milli-Q water. After ultrasonic wave treatment for 30 min, the sample was centrifuged at 6500× *g* and 4 °C for 10 min. The supernatant (8 mL) was transferred to a 20 mL vial containing 2 μL of 4-methyl-2-pentanol (125.0 mg/L) as an internal standard and 3 g of sodium chloride. Volatile compounds were collected for 45 min at 50 °C, followed by GC-MS analysis [26]. Volatile compounds were identified by matching with the NIST 14 mass spectral database and were quantified with 4-methyl-2-pentanol as an internal standard.

### 2.5. DNA Extraction and Shotgun Metagenomic Sequencing

Samples from each time point were thoroughly mixed, and then 5 g of each fermented grain sample was placed in 30 mL of phosphate-buffered saline (120 mM, pH = 8.0) and then shaken at 200× *g* for 30 min. A series of centrifugation steps were executed to enrich the microbial cells in the samples. First, the suspension was centrifuged at 200× *g* for 5 min, and the pellet was washed twice with phosphate-buffered saline. Microbial cells in the suspension were collected by centrifugation at 9000× *g* for 10 min to pellet microbial cells, followed by three washing steps.

DNA was isolated using a CTAB-based method. The microbial pellet of each sample was resuspended using 1 mL of CTAB lysis buffer (0.1 M of Tris-HCl, 0.1 M of EDTA, 0.1 M of Na_3_PO_4_, 1.5 M of Na Cl, 1% *w*/*v* CTAB (pH = 8), 5 μL of proteinase K (20 mg/mL), 10 μL of lysozyme (50 mg/mL), 10 μL of lyticase (10 u/mL), and 5 μL of RNase A (10 mg/mL)). The samples were then transferred to bead-beating tubes containing 0.3 g glass beads (0.3 mm diameter) and were homogenized using a Mini-Beadbeater twice for 2 min. After this, the samples were incubated at 37 °C for 1.5 h under horizontal shaking at 200× *g*. Two hundred microliters of 20% sodium dodecyl sulfate (SDS) was added to each sample, followed by incubation at 65 °C for 1 h while gently inverting each tube every 20 min. DNA was extracted using two extraction steps with chloroform-isoamyl alcohol (24:1 *v*/*v*) and was precipitated for 1.5 h using 0.6 volumes of pre-cooled isopropanol. Each DNA pellet was washed thrice using pre-cooled 70% ethanol. DNA was eluted using 30 μL of ultra-pure water (Milli-Q).

Sequencing was performed at Majorbio Bio-Pharm Technology Co., Ltd. (Shanghai, China). Sequence libraries were fragmented to an average size of approximately 300 bp using a Covaris M220 ultrasonicator device (Gene Company Limited, Beijing China) and were tagged with sequencing adapters using a TruSeq DNA Sample Prep Kit (Illumina, San Diego, CA, USA) according to the manufacturer’s instructions. Paired-end sequencing was performed on an Illumina HiSeq 4000 platform (Illumina).

### 2.6. Sequence Assembly, Taxonomic and Functional Annotation

To improve the accuracy of subsequent analysis, the adapter sequences were trimmed off on the 3′- and 5′-ends using the SeqPrep software. Sickle software was used to remove low-quality reads shorter than 50 bp with a quality value below 20 or with ambiguous base calls. Clean reads were assembled into contigs using the MEGAHIT software and multiple k-mer lengths [27]. The contigs shorter than 300 bp were excluded. Putative open reading frames (ORFs) were predicted using MetaGene and were clustered using CD-HIT with a 95% sequence identity and 90% coverage [28]. A non-redundant gene catalog was compiled from each cluster. Sequences were aligned against a non-redundant gene catalog using SOAPaligner at a 95% sequence identity to assess the gene abundance [29], and the gene abundance in each sample was calculated and normalized via the Reads Per Kilobase of per Million mapped reads (RPKM) [30]. The sequence data were deposited in the NCBI Sequence Read Archive under the project accession number PRJNA630248.

Taxonomic annotation was performed against the NCBI nr database using DIAMOND (http://www.diamondsearch.org/index.php, version 0.8.35) BLASTp with an e-value threshold of 1e^−5^ [31]. The EggNOG (version 4.5) [32], CAZy (version 5.0) [33], and KEGG (https://www.genome.jp/kegg/) databases [34] were employed for functional annotation using the same threshold value. The KOBAS 2.0 software was used to conduct a KEGG pathway mapping analysis [35]. The functional genes (i.e., enzyme-encoding genes) associated with fermentation were annotated, and the relative abundance of functional microorganisms at different times during fermentation was calculated using the total relative abundance of certain functional genes at different times as 100%.

### 2.7. Statistical Analyses

Data were tested by conducting a one-way ANOVA followed by Duncan’s test using SPSS (version 20.0; SPSS, Chicago, IL, USA). *p*-values < 0.05 were considered significant. A principal component analysis (PCA) was performed using the SIMCA software (version 14.0; Umetricus AB, Umea, Sweden). The microbial communities at species level in fermented grain were visualized using Circos (version 0.69–6). The orthogonal projection to latent structure discriminant analysis model (O2PLS) was used to estimate the relationship between the microbial species and polar water-soluble compounds and volatile compounds, respectively, which consisted of the simultaneous projection of both the microbial species (X) and metabolites (Y) during *Baijiu* fermentation with SIMCA-14.0 [36]. The correlation matrix shows the pair-wise correlation between all variables (X and Y). The microbial species with variable importance in the projection (VIP) value > 1.0, were the most associated for explaining the metabolites. The significance of correlation coefficient was calculated via Origin 8.0 (OriginLab Cor., Northampton, MA, USA). Only the significant correlations of data were presented and discussed. A high correlation coefficient (|ρ| ≥ 0.8, *p* < 0.05) between the microbial species (VIP > 1) and metabolites was visualized via Cytoscape (v.3.4.0). Heatmaps of the gene abundance annotated using eggNOG and the KEGG database were produced using the pheatmap package with Z-score normalization in R [37].

## 3. Results

### 3.1. General Investigation of Microbiota and Physicochemical Properties

**Figure 1:**
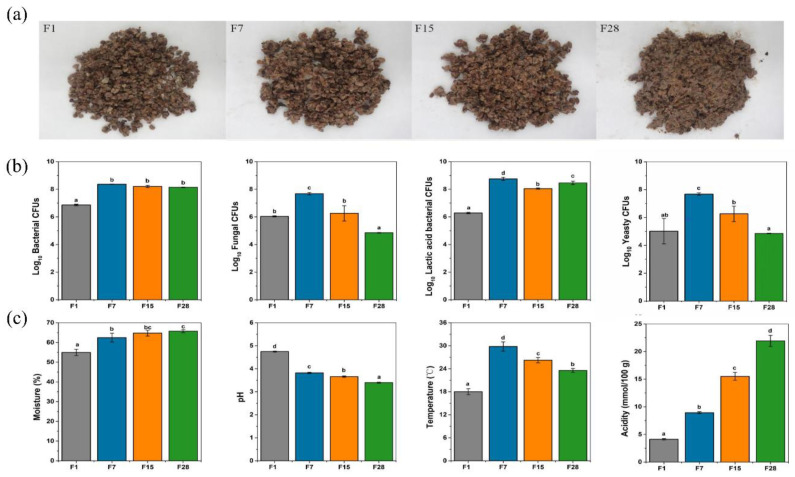
Microbiological and physicochemical characteristics of the fermented grains on the 1st, 7th, 15th, and 28th days of light-flavor *Baijiu* fermentation. (**a**) Exemplary images of fermented grains; (**b**) changes in the viable microbial counts of fermented grains; (**c**) changes in the physicochemical characteristics of fermented grains. F1: fermented grains sampled on the 1st d; F7: fermented grains sampled on the 7th d; F15: fermented grains sampled on the 15th d; F28: fermented grains sampled on the 28th d. Different letters obtained by one-way ANOVA followed by the Duncan’s test indicated significant differences at *p* < 0.05.

The culture-based microbial abundance estimates and physicochemical properties of fermented grain provided the first insights into the microbial composition and metabolic changes, which offered a basis for further omics analyses. During light-flavor *Baijiu* fermentation, the grain was transformed from loose, large sorghum particles to a watery, sticky substance (Figure 1a). The total numbers of bacteria, fungi, LAB, and yeasts were 6.86, 6.04, 6.29, and 5.01 log CFU/g, respectively, on day 1. All of these groups reached their maximum abundance by day 7. No molds were detected after day 7. The total population of bacteria was higher than that of yeasts, at approximately 3 log CFU/g on day 28 (Figure 1b). The physicochemical properties indicated the progress of the fermentation and reflected the quality of the fermented grains [38]. During fermentation, the moisture content and acidity continually increased, while the pH decreased (Figure 1c). The temperature increased rapidly from the start of the fermentation to day 7 and then decreased gradually to 24 °C on day 28.

### 3.2. Microbial Composition and Dynamics Based on Shotgun Metagenomics

**Figure 2:**
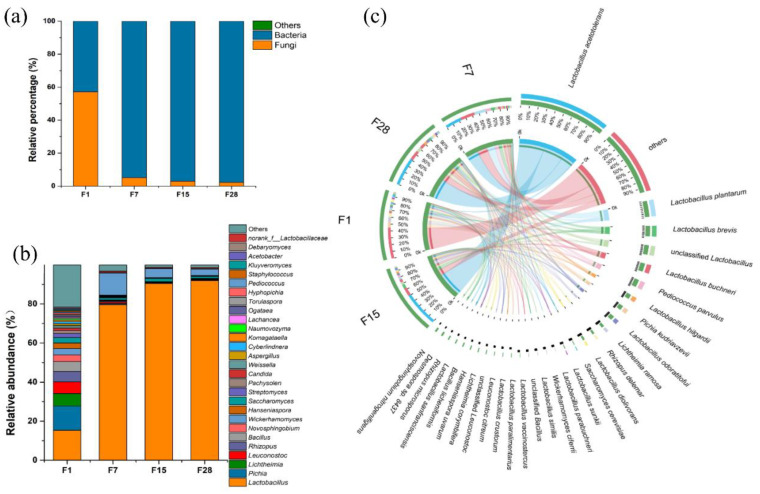
Taxonomic annotation of the microbiota in fermented grain during light-flavor *Baijiu* fermentation. (**a**) Distribution of microorganisms at the domain level; (**b**) distribution of dominant microbial genera (only genera which occurred at >0.5% in at least one sample were shown); (**c**) distribution of dominant microbial species (only species which occurred at >1% in at least one sample were shown). The green bars at the outer ring represent different samples (F1, F7, F15, and F28, at the right side of the diagram), while others represent for different species. The length and number of the bars on the inner ring represent the percentage of species in each sample. The bands with different colors demonstrate the sources of different species.

The sequencing of metagenomic libraries from the microbiota of fermented grain samples collected on days 1, 7, 15, and 28 produced a total of 539 million clean reads with each sequencing depth of 10 Gb. Details on the numbers of reads per sample are shown in Table S1. Taxonomic annotation was performed to reveal the microbial diversity during *Baiiju* fermentation. Shotgun metagenomics can help examine relative abundances at the domain level of microbial communities, which would reflect variations in the microbial populations [39]. At the start of the fermentation process, the abundance of fungi and bacteria was 58.23% and 41.70%, respectively (Figure 2a). The relative abundance of bacteria rapidly increased to 94.56% until day 7 and remained predominant until the end of the fermentation process (97.65%), whereas that of fungi decreased to 2.30%. Microbial diversity decreased sharply during fermentation. At a relative abundance of >1%, we observed 70 genera and 136 species at the beginning of the fermentation, and only 12 genera and 53 species at the end of the fermentation (Table S2). *Lactobacillus*, *Pichia*, *Lichtheimia*, *Leuconostoc*, *Rhizopus,* and *Bacillus* were predominant on day 1 and, among these, only *Lactobacillus* and *Pichia* showed a relative abundance of >10%. The relative abundance of *Lactobacillus* had increased to 79.62% by day 7, and it became the most abundant taxon until the end of fermentation, accounting for 92.02% of the total abundance at the genus level (Figure 2b). At the species level, *p. kudravzerii*, *Li. ramosa*, *Le. citreum,* and *B. licheniformis* were predominant on day 1. *La. acetotolerans*, *La. buchneri*, and *La. hilgardii* increased and were predominant at the end of the fermentation (Figure 2c). *Saccharomyces cerevisiae*, which was the main producer of ethanol, occurred at a relative abundance of 6.68% to 62.26% of all fungi from the beginning to the end of the fermentation (Table S3).

### 3.3. Metabolic Succession during Light-Flavor Baijiu Fermentation

**Figure 3:**
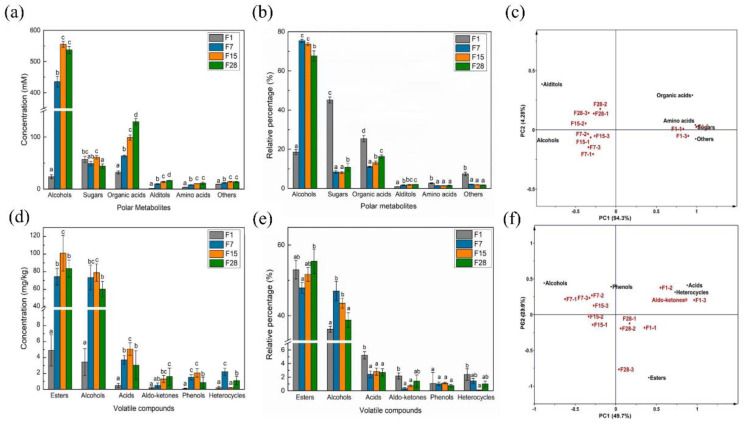
Dynamics of metabolites in the fermented grain in light-flavor *Baijiu* fermentation, including (**a**) the concentration and (**b**) relative abundance of six categories of polar water-soluble compounds detected by ^1^H NMR; (**d**) the concentration and (**e**) relative abundance of six categories of volatile compounds detected by GC-MS; a principal components analysis of (**c**) polar water-soluble compounds and (**f**) volatile compounds. Different letters obtained by one-way ANOVA followed by Duncan’s test indicated significant differences at *p* < 0.05.

Metabolites in fermented grain produce the aroma of *Baijiu*, either directly or as a precursor, and the succession of metabolites is affected by the microbiota changes during fermentation [21]. To gain a broad understanding of the metabolite changes in fermented grains during fermentation, both polar water-soluble metabolites and volatile compounds were analyzed. A total of 54 polar water-soluble metabolites, including carbonic and nitrogen compounds, was annotated at different fermentation stages, including 4 alcohols, 6 sugars, 23 organic acids, 3 alditols, 6 amino acids, and 12 other compounds (Table S4). Among these six categories, the concentrations of alcohols and amino acids increased from day 1 to day 15; organic acids and alditol increased throughout the fermentation process, whereas sugars increased until day 15 and then decreased until day 28 (Figure 3a). Sugars and organic acids were the main metabolites at the beginning of fermentation, with relative abundances of 45.14% and 25.38%, respectively; however, these abundance substantially decreased to 18.43% and 11.04% (*p* < 0.05) by day 7. Meanwhile, alcohols were the predominant compounds from day 7 to day 28, at an abundance of 67.67% (Figure 3b). Based on the profile of these compounds, a principal components analysis (PCA) plot was produced to characterize the different fermentation periods of fermented grain (Figure 3c). The first two principal components (PC1 and PC2) explained more than 85% of the total variance. The fermented grain collected on day 1 was separated from all the other samples, suggesting that the most substantial changes in the polar metabolites occurred during the first seven days.

Furthermore, 112 volatile compounds were identified and quantified in fermented grains, including 42 esters, 27 alcohols, 16 acids, 13 aldo-ketones, 5 phenols, and 9 heterocyclic compounds (Table S5). The concentrations of esters, alcohols, acids, and phenols peaked on day 15 and gradually decreased until day 28. The aldo-ketone concentrations continuously increased, and those of heterocycles fluctuated during fermentation (Figure 3d). Esters were the main volatile compounds during fermentation, with relative abundances ranging from 52.99% on day 1 to 55.39% on day 28. The relative abundance of alcohols increased until day 7 and then decreased during the remaining time period. The concentrations of acids, aldo-ketones, phenols, and heterocycles were reduced by day 7 and then remained relatively constant until the end of fermentation (Figure 3e). A PCA analysis was conducted to relate different times of fermentation to concentrations of volatile compounds. The first and second principal components (PC1 and PC2) explained 49.7% and 23.9% of the total variance, respectively. The biplot suggested that alcohols, esters, and acids were responsible for the separation of fermented grains at different fermentation stages (Figure 3f).

### 3.4. Correlations of Microorganisms and Metabolites in Fermented Grain

**Figure 4:**
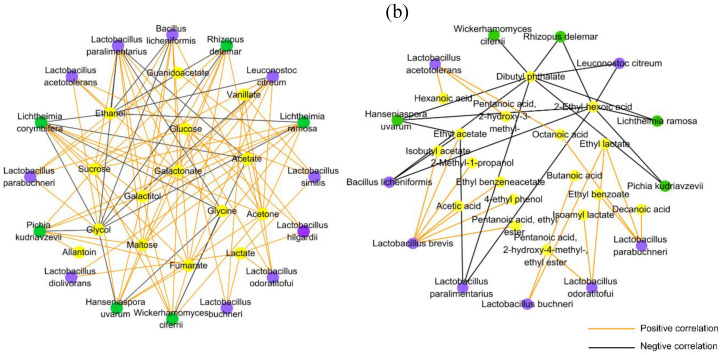
Correlation analysis between the microbial species (VIP > 1) and metabolites by the O2PLS-DA model during *Baijiu* fermentation. The significant correlated network (|ρ|  ≥  0.8, *p* < 0.05) between microbial species and (**a**) polar water-soluble compounds (**b**) and volatile compounds. The yellow nodes represent the metabolites, the purple nodes represent the bacterial species, and the green nodes represent the fugal species.

To explore the microbial functions during fermentation, the O2PLS-DA model was constructed for the correlation analysis between microbial species and metabolites, including both polar water-soluble compounds and flavor compounds (Tables S6 and S7). The R^2^Y and Q^2^ of the polar and water-soluble compounds model were 0.973 and 0.981, respectively, and those of the volatile compounds were 0.888 and 0.705, respectively. The VIP of microbial species is displayed in Figure S1. A total of 139 significant correlations (correlation coefficient ≥ |0.8|, *p* < 0.05) between microbial species (VIP > 1) and metabolites are displayed in Figure 4. Most LABs showed positive correlations with the metabolites; for instance, *La. acetotolerans* and *La. parabuchneri* were positively correlated with ethanol production; *La. acetotolerans*, *La. buchneri*, *La. odoratitofui*, *La. hilgardii*, *La. similis*, and *La. parabuchneri* showed a positive correlation with glucose and lactate; *La. odoratitofui*, *La. Hilgardii*, and *La. diolivorans* were positively correlated with acetate (Figure 4a). *La. brevis* was positively correlated with ethyl acetate, isobutyl acetate, pentanoic acid, ethyl ester, ethyl benzeneacetate, 2-methyl-1-propanol, and 4-ethyl phenol (Figure 4b), whereas *La. paralimentarius* exhibited negative correlations with most metabolites. Most fungi showed negative correlations with the majority of metabolites, apart from *Pichia kudriavzevii Li. ramose*, *Rhizopus delemar*, and *Hanseniaspora uvarum*, which produced positive correlations with sucrose, galactonate, galactitol, maltose, and acetone.

### 3.5. Functional Gene Categories of Microbiota in Fermented Grain

**Figure 5:**
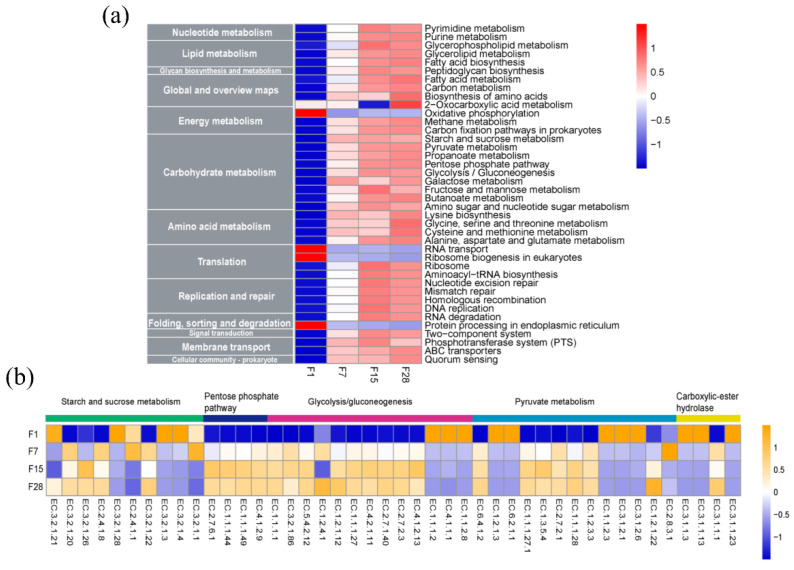
Functional gene analysis of the fermented grain microbiota in light-flavor *Baijiu* fermentation based on metagenome annotation. (**a**) Changes in the function distribution as annotated using the KEGG database; (**b**) changes in the enzyme-encoding genes as annotated using KEGG within the carbohydrate metabolism category. Heatmap is scaled by the relative abundances for each row (**a**) and column (**b**), ranging from low relative abundance to high relative abundance.

To interpret the observed correlation between the microorganisms and metabolites, the distribution of metabolic functional genes in fermented grain was annotated using the COG, CAZy, and KEGG databases, respectively. Regarding functional prediction, 1,847,233 ORFs were found, and the total and average lengths of these genes were 875 Mbp and 473.57 bp, respectively. Within the COG category related to metabolism, the relative abundance of carbohydrate, amino acid, lipid, and nucleotide transport and metabolism showed an increasing trend from the beginning of the fermentation process until day 7, and then remained stable until the end of the experiment (Figure S2). According to the CAZy database annotation, the abundance of genes associated with carbohydrate esterase, glycoside hydrolases, glycosyl transferases, and carbohydrate-binding modules increased from the beginning of the fermentation until day 7, while the auxiliary activities and polysaccharide lyases decreased. After day 7, the respective abundance of all the enzyme families was constant or decreased slightly (Figure S3). The metabolic pathways and potential functional enzymes were annotated by KEGG pathway mapping. The functional genes in fermentation were divided into 13 categories at level 2, and carbohydrate metabolism was found to be the most abundant category (Figure 5a). The majority of genes for carbohydrate metabolism increased by day 15 and then remained constant until the end of fermentation. The enzymes in the carbohydrate metabolism categories, including starch and sucrose metabolism, pentose phosphate pathway, glycolysis/gluconeogenesis, pyruvate metabolism, and some carbolic-ester hydrolases, are essential for *Baijiu* fermentation [40]. Within the category of starch and sucrose metabolism, the relative abundance of enzyme-encoding genes for α-amylase (EC 3.2.1.1) increased from day 1 to day 7 and then decreased until the end of the fermentation (Figure 5b). The relative abundance of genes for glucoamylase (EC 3.2.1.3) decreased throughout the fermentation process, and the relative abundance of enzyme-encoding genes for β-glucosidase (EC 3.2.1.21) decreased during the first 15 days, but slightly increased until the end of fermentation. The majority of enzymes encoding genes associated with the pentose phosphate pathway and glycolysis/gluconeogenesis showed an increasing trend during the first 15 days. Carboxylic-ester hydrolases are the most important contributors to flavor compound formation. The relative abundance of the triacylglycerol lipase (EC 3.1.1.3)-encoding genes decreased throughout the fermentation process, whereas the carboxylesterase (EC 3.1.1.1)-encoding genes generally increased.

### 3.6. Metabolic Potential of Microbiota in Fermented Grain

**Figure 6:**
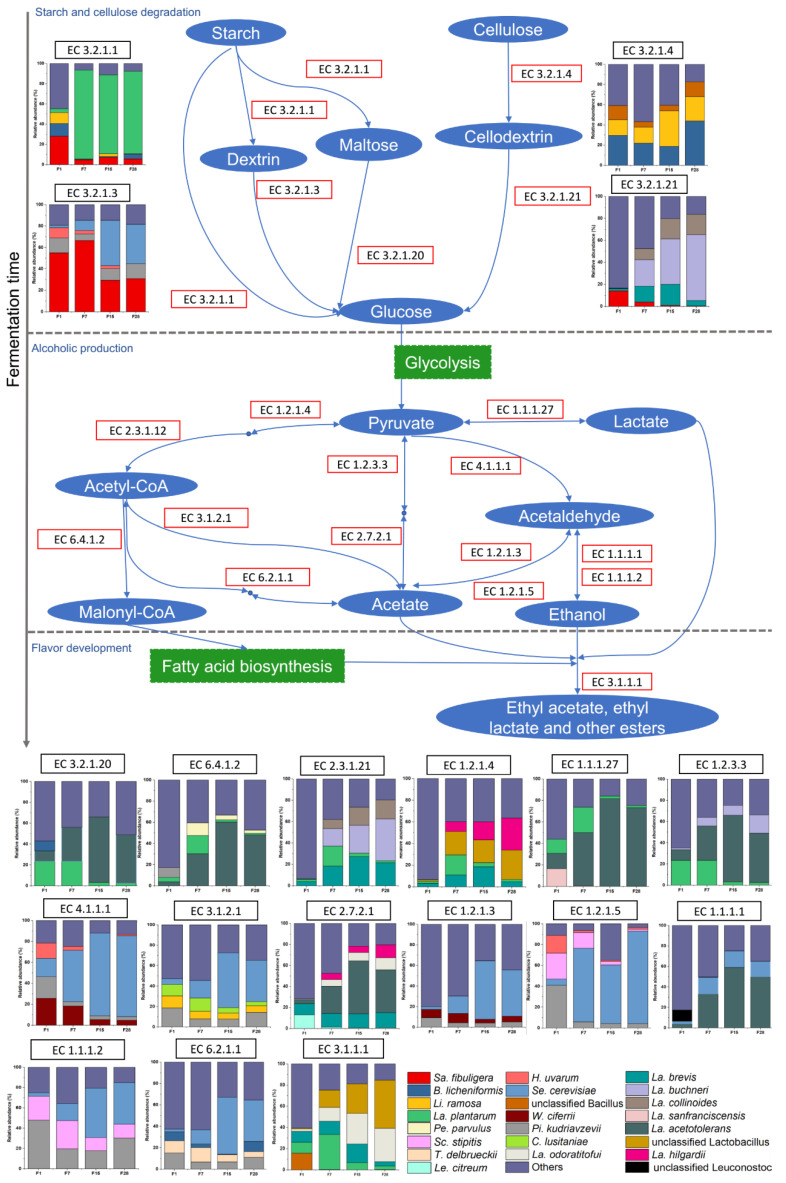
Schematic overview of the metabolic processes with dominant microorganisms during light-flavor *Baijiu* fermentation. For each enzyme, only a microbial relative abundance of >10% in at least one sample is present. “Sa”: *Saccharomycopsis*; “B”: *Bacillus*; “Li”: *Lichtheimia*; “La”: *Lactobacillus*; “Pe”: *Pediococcus*; “Sc”: *Scheffersomyces*; “T”: *Torulaspora*; “Le”: *Leuconostoc*; “H”: *Hanseniaspora*; “Se”: *Saccharomyces*; “W”: *Wickerhamomyces*; “Pi”: *Pichia*; “C”: *Candida*; “Se”: *Saccharomyces*.

To further explore the metabolic potential and the respective microorganisms during fermentation, the most abundant pathways, enzymes, and dominant microorganisms associated with the respective enzymes were identified, and a metabolic illustration was produced (Figure 6). These metabolic processes can be divided into three stages: (i) starch and cellulose degradation, (ii) alcoholic production, and (iii) flavor development. There were three main pathways for starch degradation in which α-amylase (EC 3.2.1.1) was required. The dominant microorganisms containing α-amylase genes were *Saccharomycopsis fibuligera*, *B. licheniformis*, and *Li. ramosa* at the beginning of the fermentation, and *La. plantarum* in the following days. Since sorghum is the main raw material, cellulose hydrolysis is also important during fermentation, and *B. licheniformis* and *Li. ramosa* were associated with cellulase (EC 3.2.1.4), while the dominant microorganisms producing β-glucosidase (EC 3.2.1.21) varied on day 1, with *Saccharomycopsis fibuligera* being the most abundant. After this, *La. Buchneri*, and *La. brevis* were the dominant β-glucosidase-producing microorganisms. During alcoholic fermentation, alcohol dehydrogenase (EC 1.1.1.1) and alcohol dehydrogenase (NADP^+^) (EC1.1.1.2) were the most abundant enzymes for ethanol production. The predominant species producing these enzymes were *La. acetotolerans*, *Saccharomyces cerevisiae*, and *P. kudriavzevii*. In addition, *P. kudriavzevii* also primarily participated in acetaldehyde formation, which was a precursor of ethanol. For instance, *La. plantarum*, *La. acetotolerans*, and *La. brevis* contributed to L-lactate dehydrogenase (EC 1.1.1.27) and acetate kinase (EC 2.7.2.1), which are required for lactate and acetate formation. After the accumulation of the precursors, flavor compounds were produced. The dominant genera producing carboxylesterase (EC 3.1.1.1) were *Lactobacillus*, including *La. plantarum* and *La. brevis* at the early stage, and *La. odoratitofui* from day 7 to day 28.

## 4. Discussion

Optimizing the fermentation process of food products is challenging because of the complex composition and succession of the microbiota [1,2,21]. Unraveling the functionality of the microbiota is fundamental to manipulating the community so as to produce the desired outcome, and this is the theoretical basis of solid-state fermentation mechanisms [22,40]. Our results contribute to an understanding of the microbial mechanisms during solid-substrate *Baijiu* fermentation and offer potential guidance for improving *Baijiu* quality via fermentation.

Coupling metagenomics with plate-counting techniques provided a clear overview of the microbial population. Compared with fungi, the bacteria at the late stage of fermentation were dominant. Notably, metagenomic sequencing showed that bacteria represented over 90% of all the microorganisms at the end of fermentation. This was in line with the results of culture-dependent analyses (Figure 1b), which was also in agreement with the results of a previous study [41]. Microbial composition during *Baijiu* fermentation has been investigated using multiple approaches, however, most previous studies produced taxonomic identification only at the genus level [6,9,42,43]. Over the time of fermentation, the microbial diversity decreased rapidly due to environmental factors, including nutrient limitation, high ethanol concentrations, and low pH values [44], and only *Lactobacillus* and several yeasts survived until the end of the process.

Considering that microbiota drive the fermentation process, functional microorganisms—i.e., metabolically active microorganisms—and their dynamics appear to be more important than the microbial composition [21]. We combined two metabolomic approaches in order to detect metabolites to improve our understanding of the biochemical processes involved in fermentation [45]. The concentration of the majority of metabolites, such as alcohols, esters, acids, amino acids, and phenols, that accumulated in fermented grain increased during the first 15 days, and then remained stable or decreased. Moreover, based on the PCA analysis of metabolites, there was an obvious shift from day 1 to day 7. Subsequently, to reveal the metabolic functions of the microbiota at gene level, functional gene categories were identified and annotated using sequence data. Shotgun metagenomics can provide strong evidence for functional predictions [16,40]. We found that the abundance of genes associated with metabolism generally increased during the first 15 days, as annotated using different databases. Considering microbial composition dynamics, the most critical changes in both microorganisms and metabolites may be speculated to occur during the first 15th days. This finding suggests that fermentation time during the solid-state fermentation process of light-flavor *Baijiu* may be reduced.

Based on the observed relationships between microorganisms and metabolites as well as functional gene annotation associated with the metabolic potential of microbial species, it was found that lactic acid bacteria were the predominant bacteria in light-flavor *Baijiu* fermentation, particularly *Lactobacillus*. Different *Lactobacillus* species contributed to the fermentation process. *La. plantarum* provided enzyme-encoding genes for starch degradation, and some *Lactobacillus* species such as *La. buchneri* and *La. collinoides* contributed to the production of β-glucosidase (EC3.2.1.21), which is associated with cellulose degradation and completes hydrolysis by converting cellobiose to glucose [46]. In addition, β-glucosidase can improve the production of some terpenes, which enhance floral aroma characteristics [47]. *La. plantarum* was reported to produce high-yield extracellular α-amylase for starch degradation, following isolation from Nigerian fermented products [48]. At the alcohol production stage, *Lactobacillus* mainly contributed to pyruvate metabolism associated with lactic acid, acetic acid, and acetyl-CoA production, which has been well studied previously [49]. Interestingly, the genes of *La. acetotolerans* strongly contributed to alcohol dehydrogenase (EC 1.1.1.1) production, which is responsible for ethanol production during the fermentation of food products [21]. Meanwhile, *La. acetotolerans* was positively correlated with ethanol (Figure 4a). *Lactobacillus* was previously found to simultaneously produce lactate, ethanol, and acetate [50]. A high abundance of genes encoding alcohol dehydrogenases could explain why *La. acetotolerans* was predominant at the late stage of fermentation, suggesting that this species was generally tolerant to high ethanol concentrations. Specific cultures of *La. acetotolerans* could be adapted to ethanol concentrations of more than 12% (*v*/*v*) [51]. Some *Lactobacillus* species such as *La. odoratitofui*, *La. plantarum*, and *La. brevis* contributed to carboxylesterases (EC3.1.1.1), which are responsible for ester formation and are important enzymes enhancing the flavor of fermented food products [52]. Ethyl esters are the most typical flavor compounds of light-flavor *Baijiu* [53]. *La. paralimentarius* was only abundant on day 1 and then almost disappeared during fermentation (Table S3). This may explain why it produced a negative correlation with most metabolites and did not play a significantly role in gene functional annotations. These results imply that *Lactobacillus* groups with multiple functions are vital for light-flavor *Baijiu* production, but further research would be needed, including, for instance, fortified fermentation with *Lactobacillus* species but avoiding excessive acidification.

In addition to *Lactobacillus*, *B. licheniformis* is also a functional species during fermentation, and it is known for starch and degradation functions due to its prominent ability to produce α-amylase and cellulase under a wide range of pHs and temperatures [54]. Although fungi only accounted for a small proportion of the total microorganisms from day 7 day to the end of fermentation (Figure 2), they were crucial for starch degradation and alcoholic fermentation, and they provided precursors for the synthesis of flavor compounds. In particular, all the microorganisms associated with the production of glucoamylase (EC 3.2.1.3) were fungi, including *Saccharomycopsis fibuligera* and *P. kudriavzevii* (Figure 6). Furthermore, *P. kudriavzevii* contributed to acetate, acetaldehyde, and ethanol formation in the pyruvate metabolism. *Saccharomycopsis fibuligera* is a major amylolytic yeast in the food industry which can assimilate multiple carbohydrate sources such as glucose, sucrose, cellobiose, and soluble starch [55]. *P. kudriavzevii* has shown multi-stress tolerance, and it is known to produce organic acids during wine fermentation [56]. Furthermore, *P. kudriavzevii* has been reported to contribute to ester formation during *Baijiu* production [57]. However, in the current study, *P. kudriavzevii* mainly participated in the synthesis of flavor compound precursors. In addition to the main producer of ethanol, *Saccharomyces cerevisiae* played a role in the conversion of acetyl-CoA and acetaldehyde, to which flavor compound formation was attributed. *Li. ramosa* was predominant in starch degradation by the potential production of α-amylase, glucoamylase, and cellulase, which has been demonstrated in previous studies [58,59].

In conclusion, microbiomes in a light-flavor *Baijiu* fermentation were systematically investigated via the combination of shotgun metagenomics and metabolomics. The microbial structure and characteristic metabolites were examined during fermentation. The microbe–metabolite relationship was established, and the further metabolic potential of core microbial species were constructed. *Lichtheimia ramose*, *Saccharomycopsis fibuligera*, and *Bacillus licheniformis* contributed the most to starch saccharification. Yeasts, such as *Saccharomyces cerevisiae* and *Pichia kudriavzevii*, were responsible for ethanol formation. Different kinds of *Lactobacillus* species, such as *La. plantarum, La. Brevis*, and *La. odoratitofui*, contributed to fermentation and acted as the main flavor-producing microorganisms potentially during the late stage of fermentation. This work provides information for the fermentation mechanism of Chinese *Baijiu* and contributes a further step to high and uniform product quality.

## Supplementary Materials

The following are available online at https://www.mdpi.com/2076-2607/8/9/1281/s1: Figure S1: Heatmap of correlation analysis between microbial species and metabolites. Figure S2: Changes of function distribution annotated by COG database during light-flavor *Baijiu* fermentation. Figure S3: Changes of function distribution annotated by CAZy database. Table S1: Statistics of the metagenomic sequencing after quality trimming from fermented grain samples. Table S2: The number of genus and species in fermented grain detected by shotgun metagenomic sequence. Table S3: Relative abundance of dominant bacteria and fungi in fermented grain, perspectival (>1%). Table S4: Concentration of polar non-volatile metabolites in fermented grain by ^1^H NMR. Table S5: Concentration of volatile compounds in fermented grain by HS-SPME-GC-MS. Table S6: O2PLS correlation matrix and P value between microbial species and polar, water soluble compounds. Table S7: O2PLS correlation matrix and P value between microbial species and volatile compounds.
