# Supplementary material for: Composition and Metabolic Functions of the Microbiome in Fermented Grain during Light-Flavor Baijiu Fermentation
Source: Microorganisms. 2020 Aug 22;8(9):1281. doi: 10.3390/microorganisms8091281 (PMC7564364; doi:10.3390/microorganisms8091281)
Supplement: Supplementary file 1 [file microorganisms-08-01281-s001.zip › microorganisms-888580 r 1 Supplementary/microorganisms-888580-r1 Supplementary figures.docx]

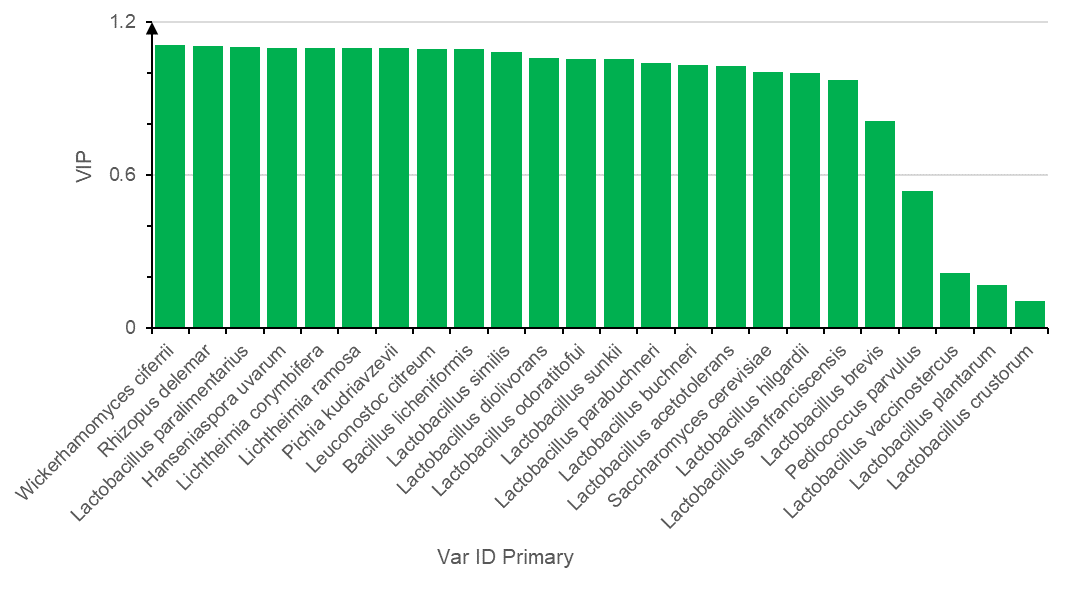


(b)

(a)


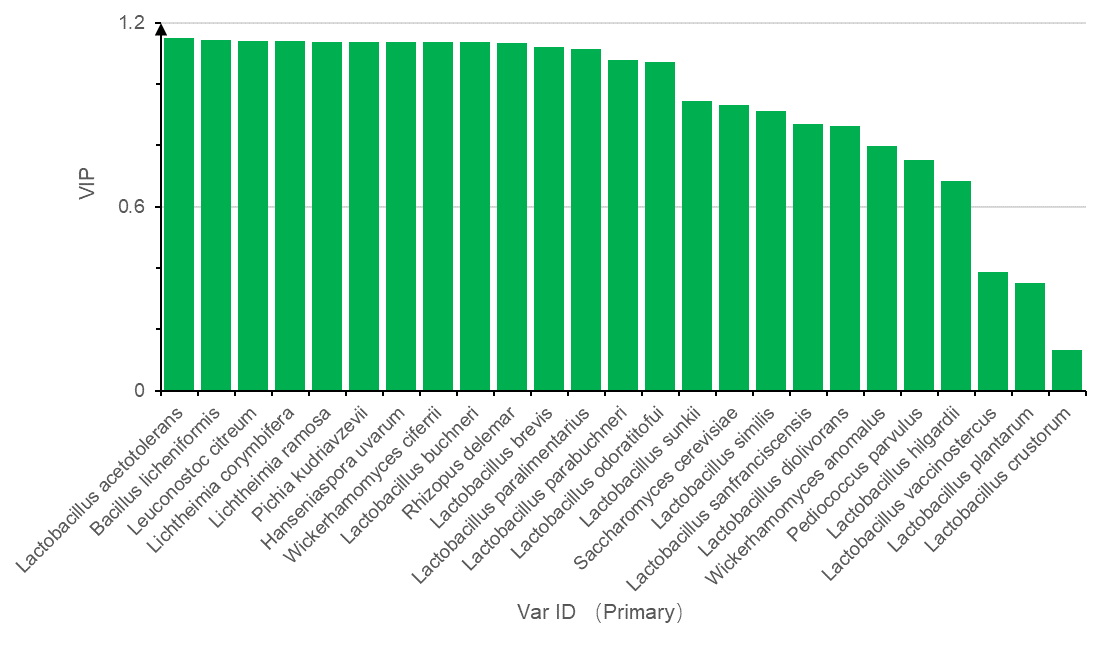


Fig. S1 Variable importance for predictive components (VIP) plot of microbial species with (a) polar, water-soluble metabolite detected by 1H NMR and (b) volatile compounds detected by GC-MS.


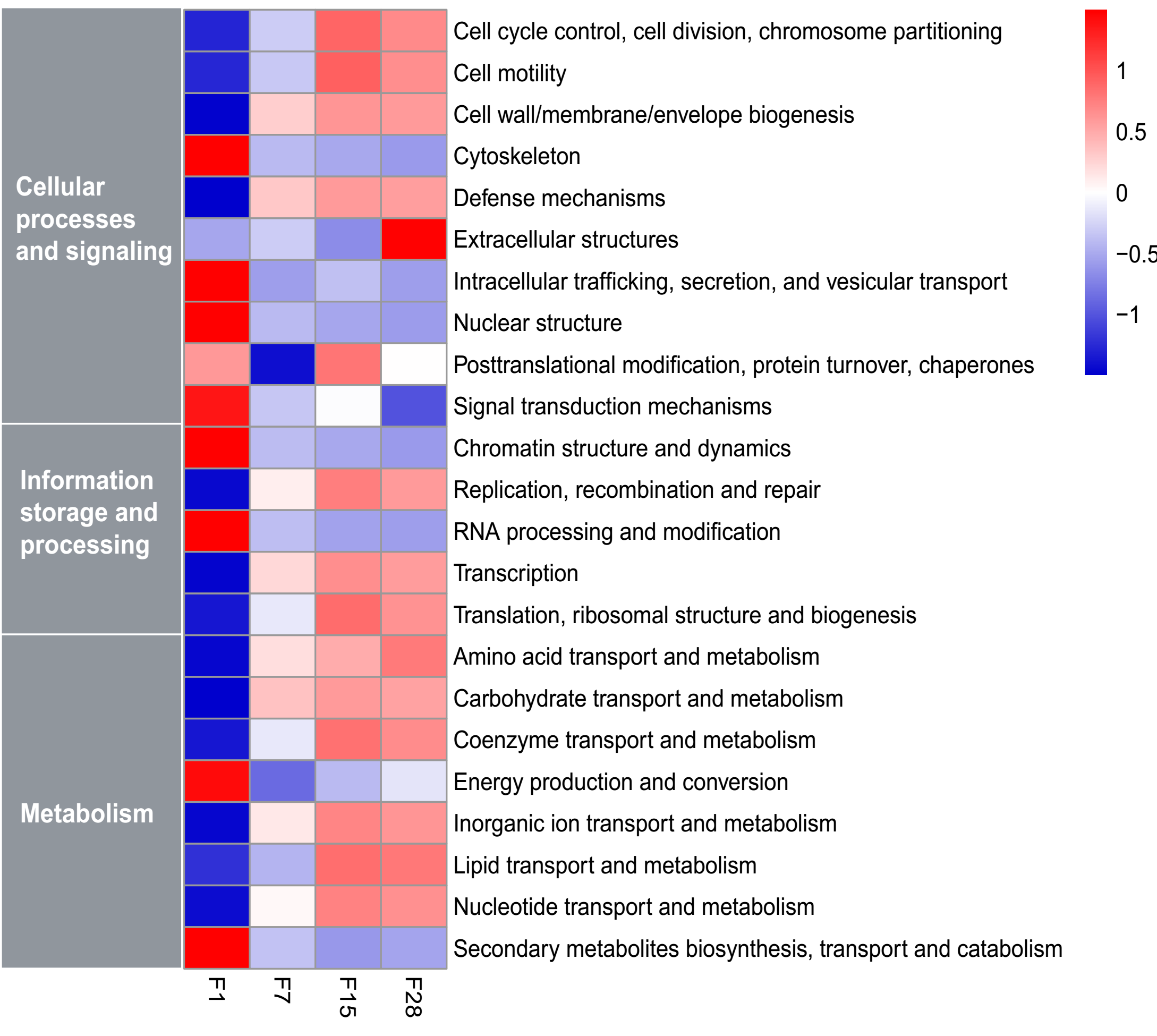


Fig. S2 Changes of function distribution annotated by COG database during light-flavor *Baijiu* fermentation.





Fig. S3 Changes of function distribution annotated by CAZy database. AA: auxiliary activities; CBM: carbohydrate-binding modules; CE: carbohydrate esterases; GH: glycoside hydrolases; GT: glycosyl transferases; PL: polysaccharide lyases;
